# Supplementary material for: Non-invasive transdermal delivery of biomacromolecules with fluorocarbon-modified chitosan for melanoma immunotherapy and viral vaccines
Source: Nat Commun. 2024 Jan 27;15:820. doi: 10.1038/s41467-024-45158-6 (PMC10821906; doi:10.1038/s41467-024-45158-6)
Supplement: Supplementary file 1 — Supplementary Information [file 41467_2024_45158_MOESM1_ESM.pdf]

Non-invasive transdermal delivery of biomacromolecules with fluorocarbon-modified chitosan for melanoma immunotherapy and viral vaccines

Wenjun Zhu[1], Ting Wei[1,2], Yuchun Xu[1], Qiutong Jin[1,2], Yu Chao[1], Jiaqi Lu[1,2], Jun Xu[1], Jiafei Zhu[1], Xiaoying Yan[1], Muchao Chen[1], Qian Chen[*1], and Zhuang Liu[*1,2]

1, Institute of Functional Nano & Soft Materials (FUNSOM), Collaborative Innovation Center of Suzhou Nano Science and Technology, Jiangsu Key Laboratory for Carbon-based Functional Materials and Devices, Soochow University, Suzhou 215123, China

2, Suzhou InnoBM Pharmaceutics Co. Ltd., Suzhou, Jiangsu, 215213, China

* zliu@suda.edu.cn, chenqian@suda.edu.cn

Supplementary Figures

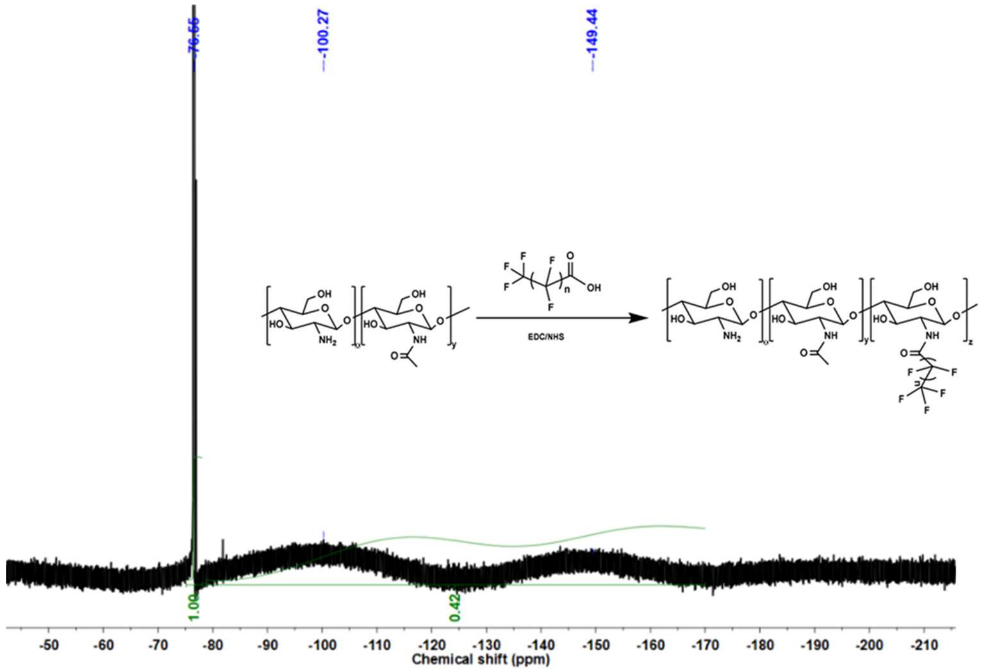

Figure S1. The 19F NMR spectra and chemical structure of FCS. Chemical shift: -76.77ppm, narrow peak, CF_3COOH(Internal standard); -100.27ppm, broad peak, -CF_3,-CF_2-CF_2-C(O)NH-;-149.44ppm, broad peak, CF_3-CF_2-CF_2-CF_2-.

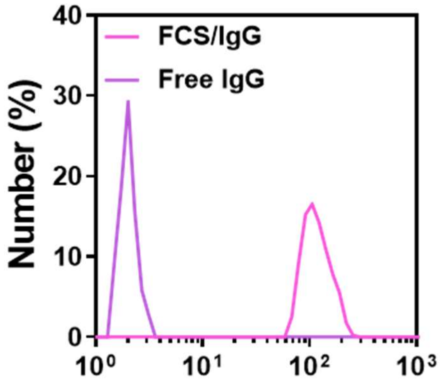

Figure S2. The DLS of FCS/IgG and free IgG

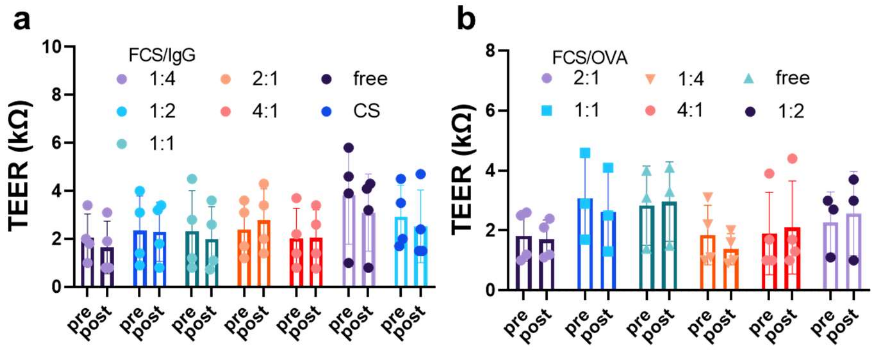

Figure S3. The skin resistances of mice skins pre and post transdermal delivery of (a) FCS/IgG and (b) FCS/OVA prepared at different FCS : protein mass ratios. Data are presented as mean ± standard deviation (n=3 or 4).

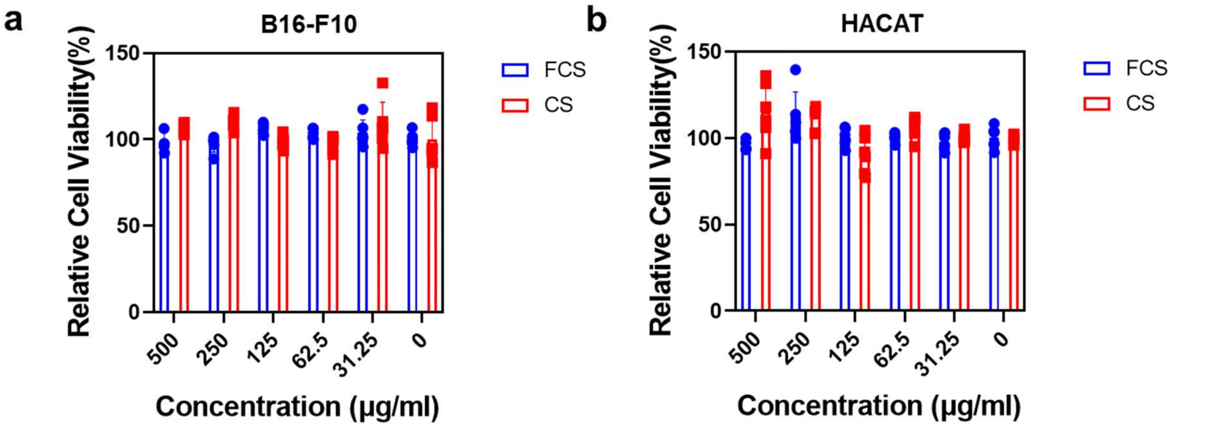

Figure S4. The cytotoxicity of FCS in B16 and HACAT cells. Data are presented as mean ± standard deviation (n=6 for both two figures).

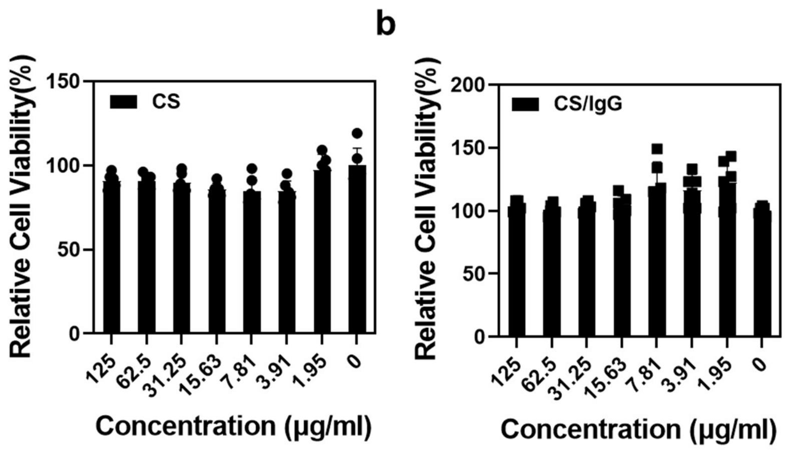

Figure S5. The cytotoxicity of (a) CS and (b) CS/IgG nanocomplexes with the concentration gradient of CS. Data are presented as mean ± standard deviation (n=6).

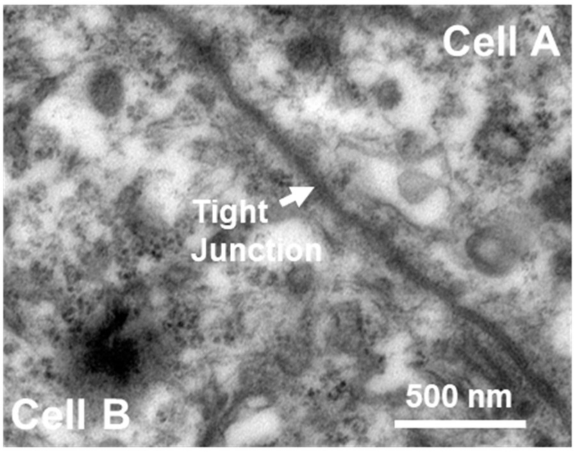

Figure S6. TEM image of normal mouse skin, showing the tight junction between epidermis (n=3).

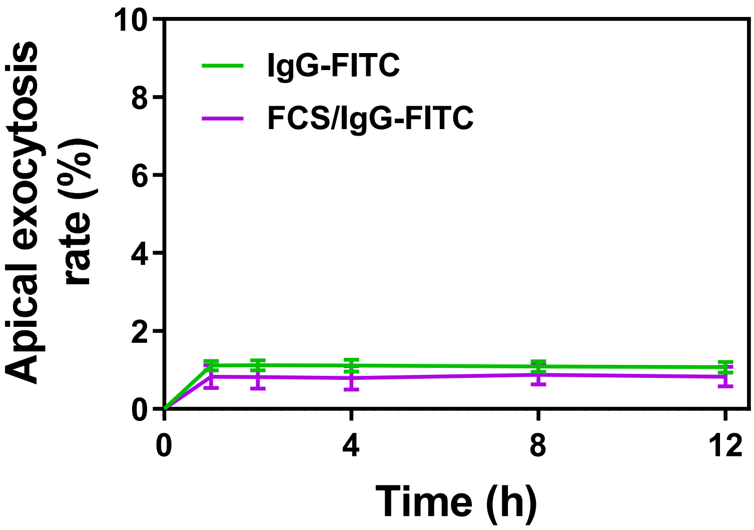

Figure S7. The apical exocytosis rate of HACAT cells after being incubated with different FCS formulations for 12 hours. Data are presented as mean ± standard deviation (n=3).

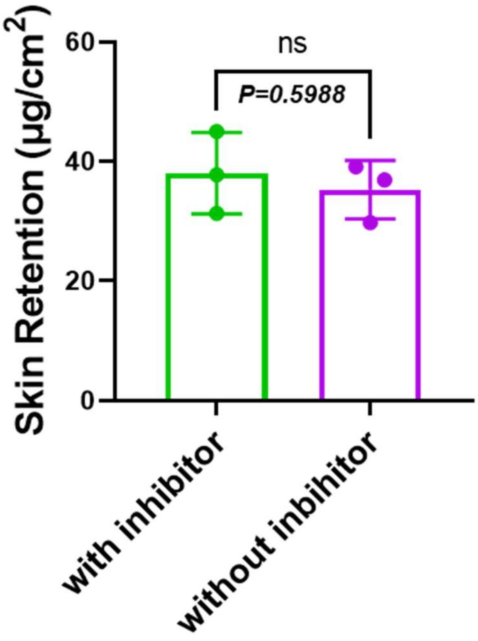

Figure S8. The skin retention FCS/IgG-FITC for 12 hours with. Skin was treated with or without 0.1 mg/mL chlorpromazine hydrochloride (the inhibitor for clathrin) for 2 hours. Data are presented as mean ± standard deviation (n=3). Statistical significance was calculated via one-way ANOVA with a Tukey post-hoc test. *P < 0.05; **P < 0.01; ***P < 0.001.

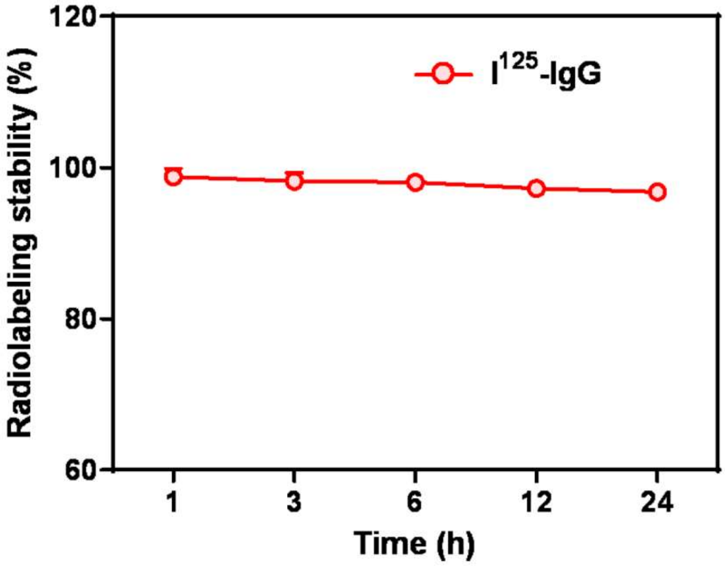

Figure S9. Radiolabeling stability of ^{125} I-IgG after incubation in serum at 37 ºC for different periods of time (n=3).

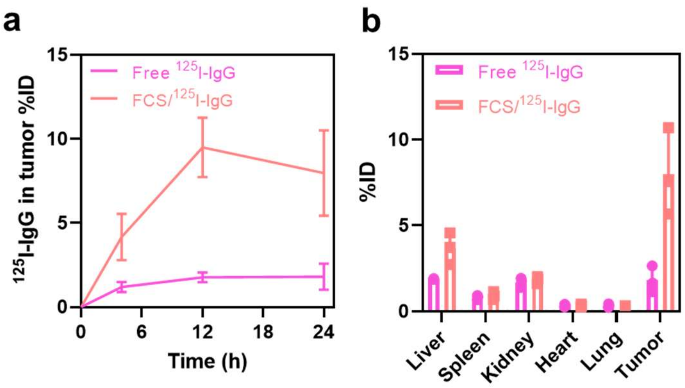

Figure S10. (a) The accumulation of FCS/^{125}I-IgG in the tumor at different time intervals. (b) Biodistribution of FCS/^{125}I-IgG at 12 h based on radioactivity measurement. Data are presented as mean ± standard deviation (n=3).

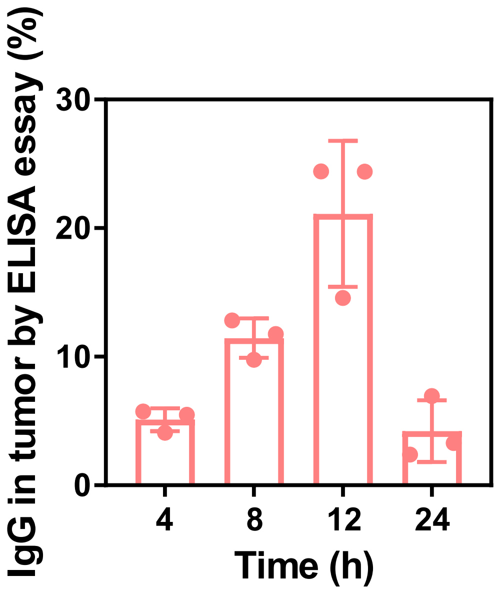

Figure S11. Statistical analysis of the accumulation of FCS/IgG in the tumors at different time points according to the ELISA essay by IgG specific binding. Data are presented as mean ± standard deviation (n=3).

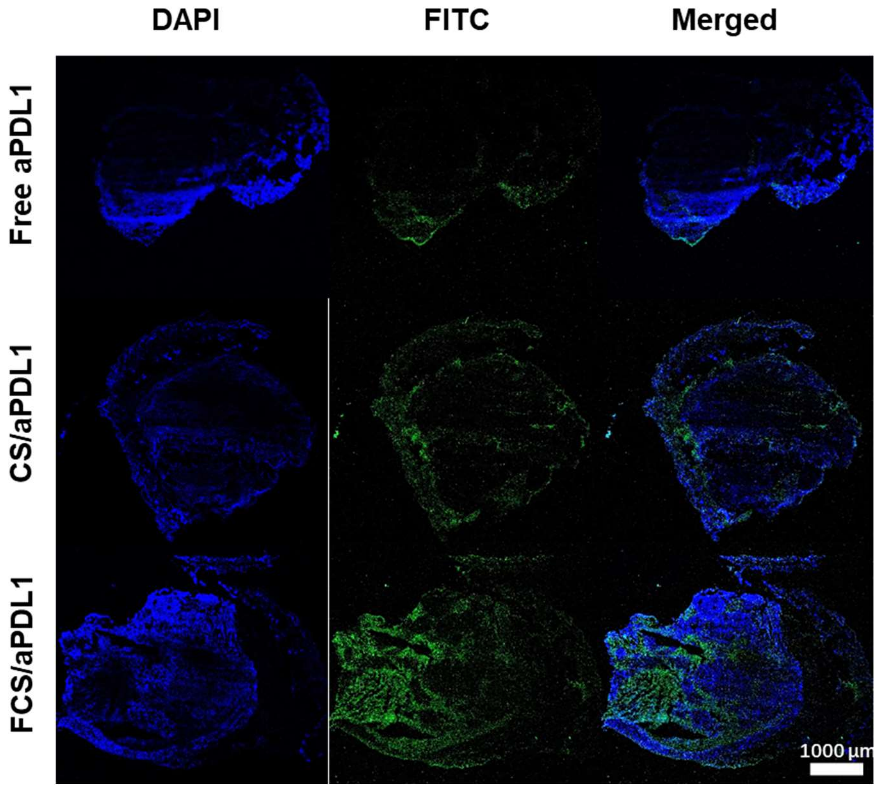

Figure S12. Representative confocal image of the transdermal accumulation of aPDL1 in tumors in 12 h. The therapeutic antibody aPDL1 was used as the primary antibody to bind to PDL1 ligand on tumor cells. The FITC labeled secondary antibody was used to specifically bind to the Fc region of aPDL1. The captured confocal image was the total size of the tumor (n=3). Scale bar: 1000 μm

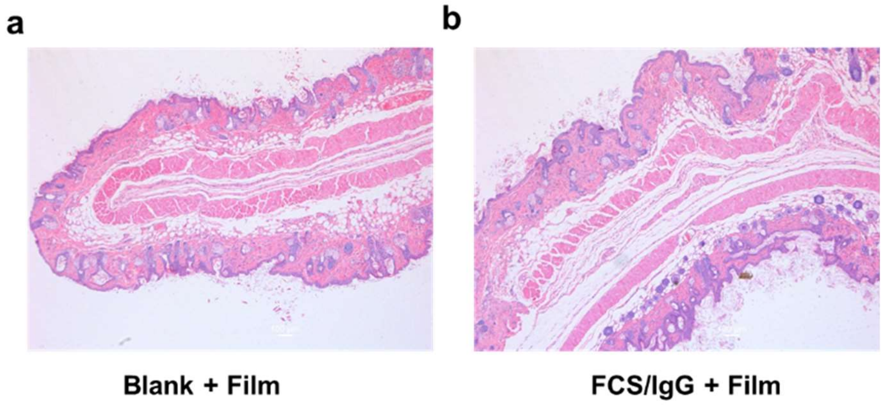

Figure S13. H&E staining of mouse skins after FCS/IgG application (n=3 for both two figures).

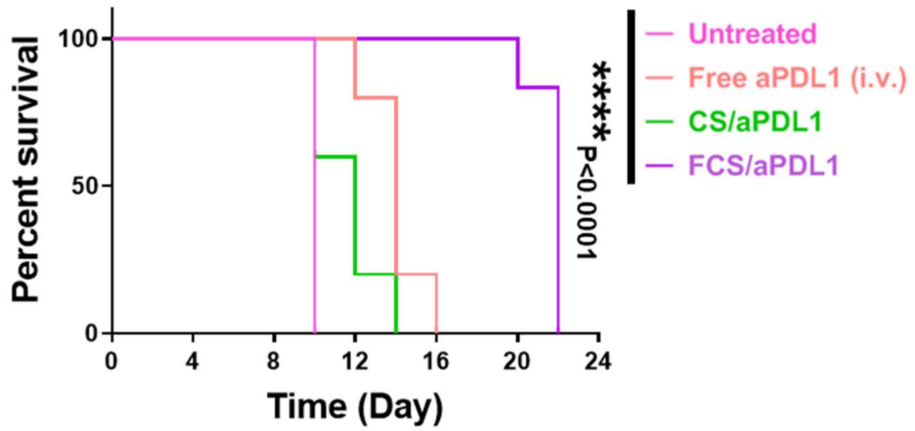

Figure S14. Survival of mice in different groups (n=5). Mice were regarded as dead when they were truly dead or their tumor size was larger than 1,000 mm^3. Statistical significance was calculated via log-rank (Mantel-Cox) test. *P < 0.05; **P < 0.01; ***P < 0.001; ****P < 0.0001.

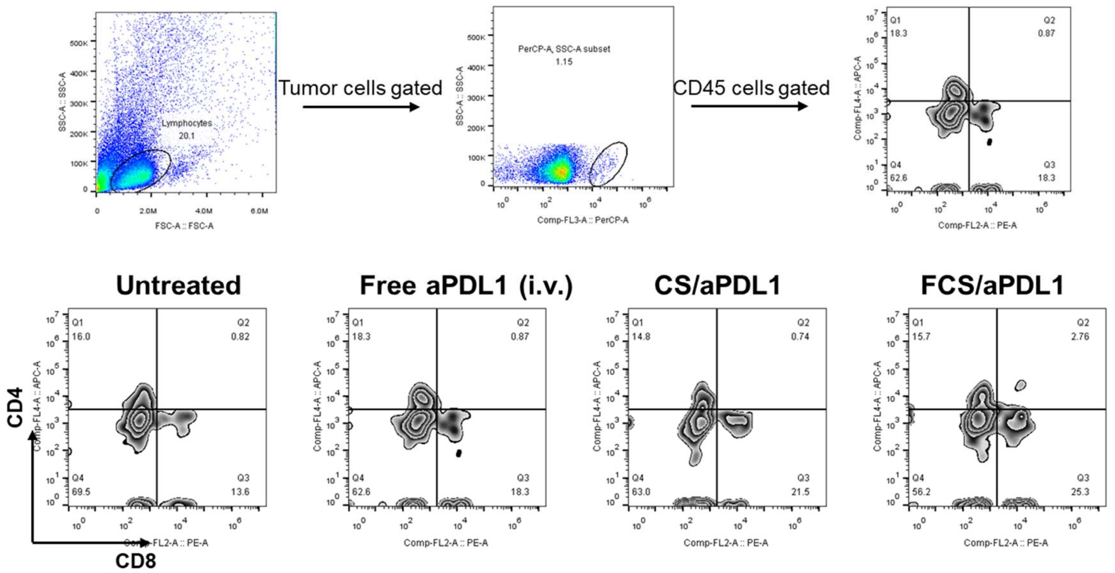

Figure S15. Representative flow cytometric plots of (Figure 3f) $CD4^+$ T cells and $CD8^+$ T cells in local tumors after different treatments.

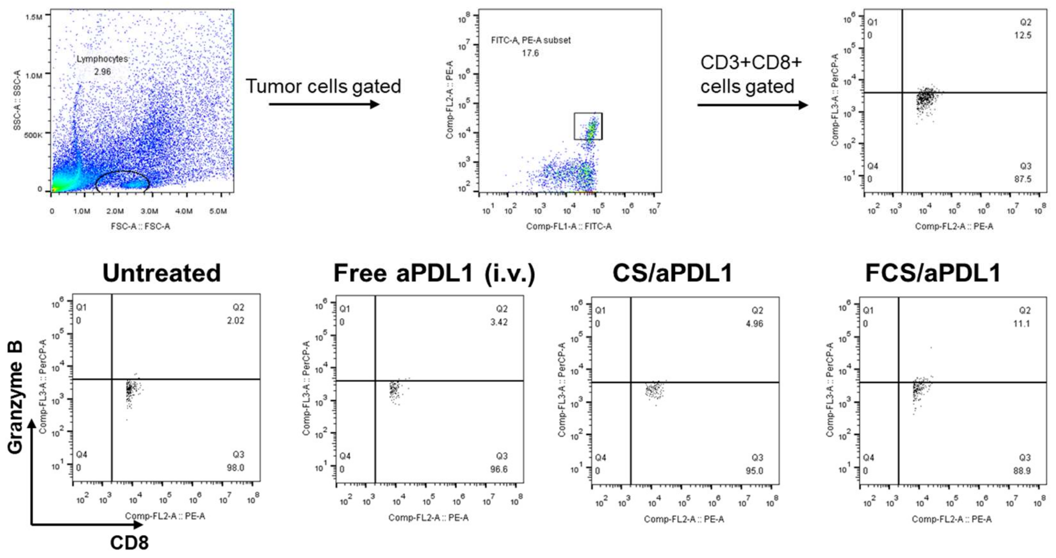

Figure S16. Representative flow cytometric plots of (Figure 3g) Granzyme B^{+}CD8^{+} T cells in local tumors after different treatments.

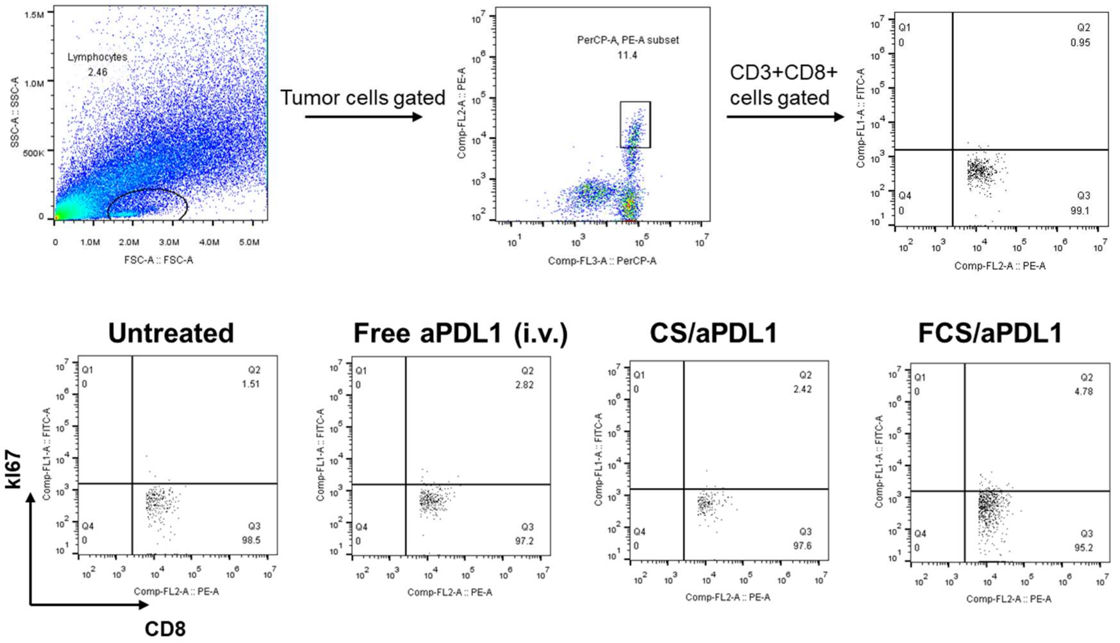

Figure S17. Representative flow cytometric plots of (Figure 3h) ki67^{+}CD8^{+} T cells in local tumors after different treatments.

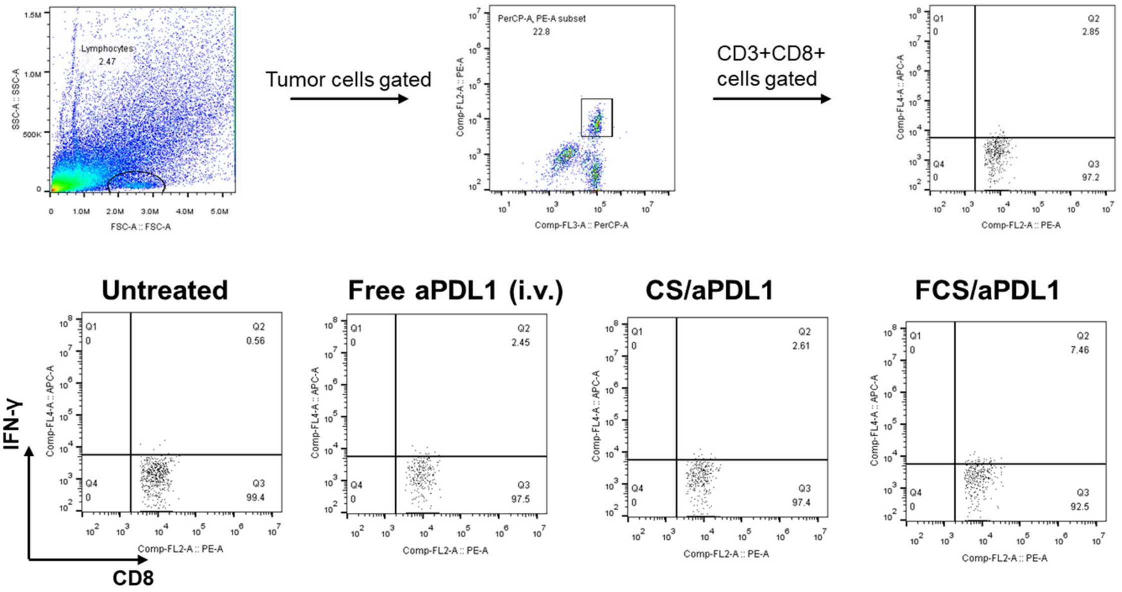

Figure S18. Representative flow cytometric plots of (Figure 3i) IFN-γ^{+}CD8^{+} T cells in local tumors after different treatments.

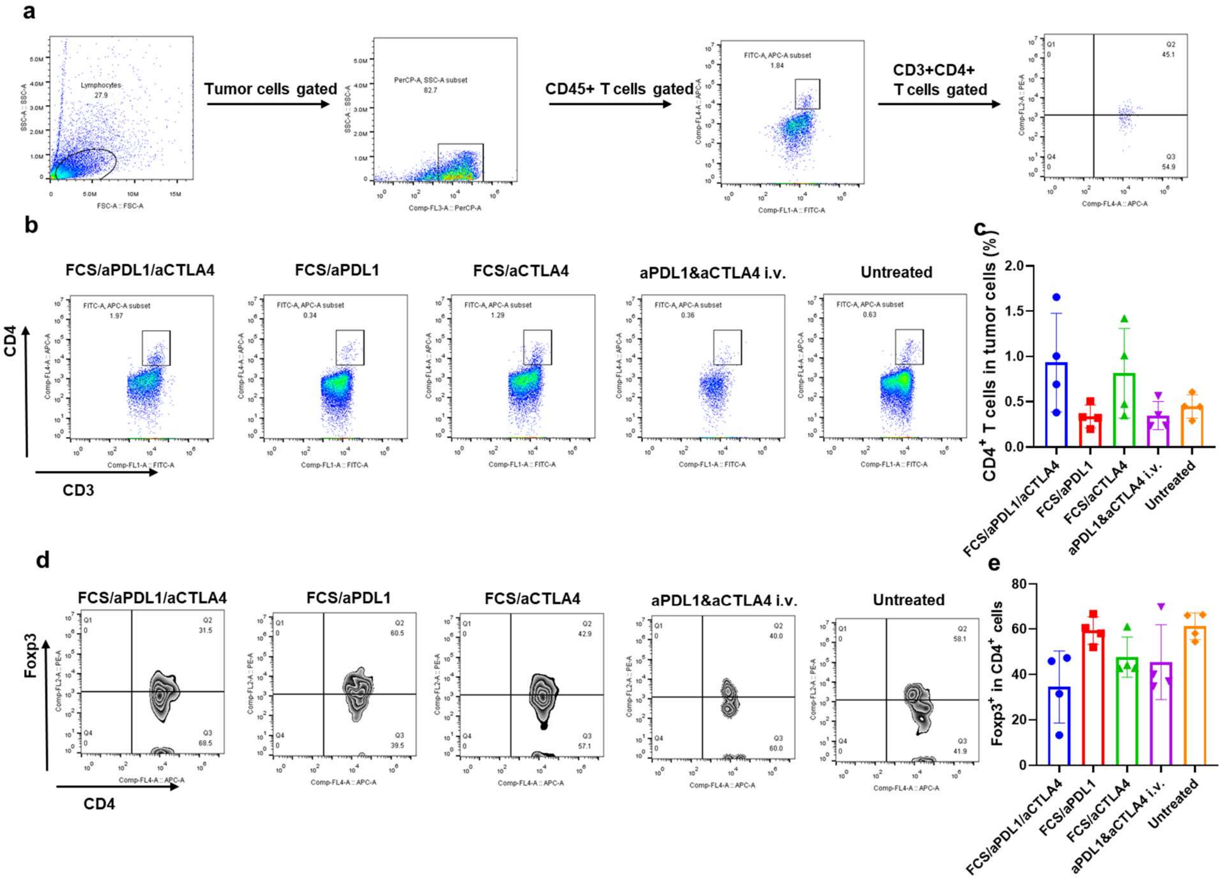

Figure S19. The analysis of CD4+ T cells in the primary tumor. (a) The gating strategy of CD4+ T cells and Tregs. (b) Representative flow cytometric plots of CD4+ T cells in CD45+ T cells. (c) Statistical analysis of CD4+ T cells in the tumor cells. (d) Representative flow cytometric plots of regulatory T cells (Foxp3+) in CD4+ T cells. (e) Data analysis of regulatory T cells (Foxp3+) in CD4+ T cells. Data are presented as mean ± standard deviation (n=4).

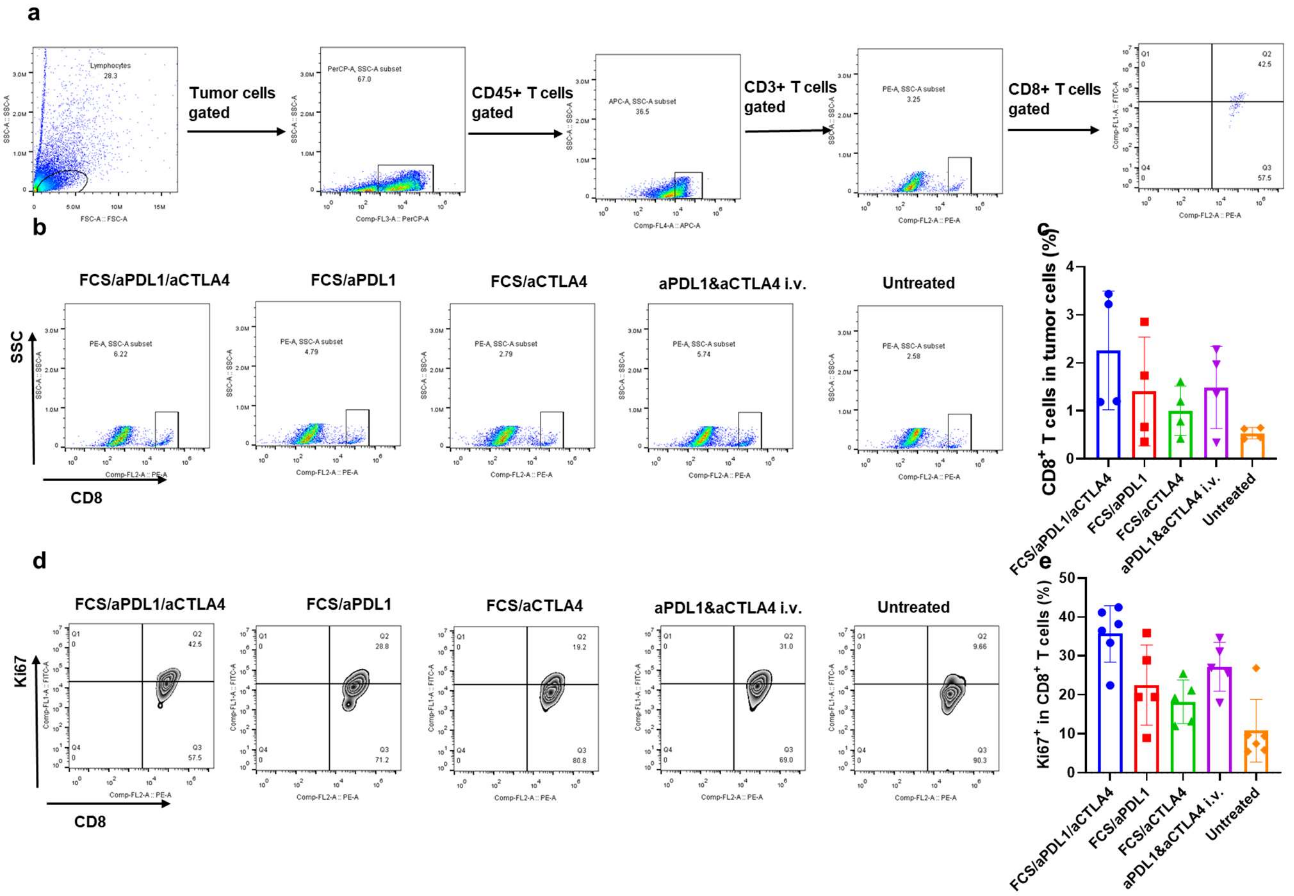

Figure S20. The analysis of CD8+ T cells in the primary tumor. (a) The gating strategy of CD8+ T cells. (b) Representative flow cytometric plots of CD8+ T cells in CD45+ T cells. (c) Statistical analysis of CD8+ T cells in the tumor cells. (d) Representative flow cytometric plots of cytotoxic CD8+ T cells (Ki67+) in CD8+ T cells. (e) Data analysis of cytotoxic CD8+ T cells (Ki67+) in CD8+ T cells. Data are presented as mean ± standard deviation (n=4).

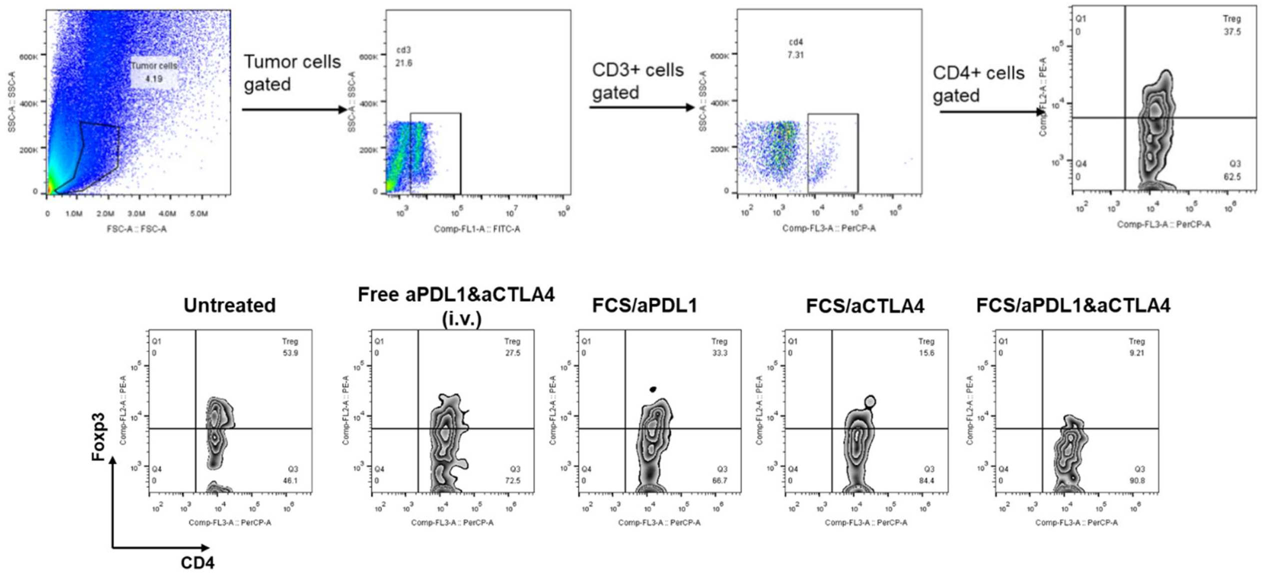

Figure S21. Representative flow cytometric plots of (Figure 4j) regulatory T cells in the distant tumors after different treatments.

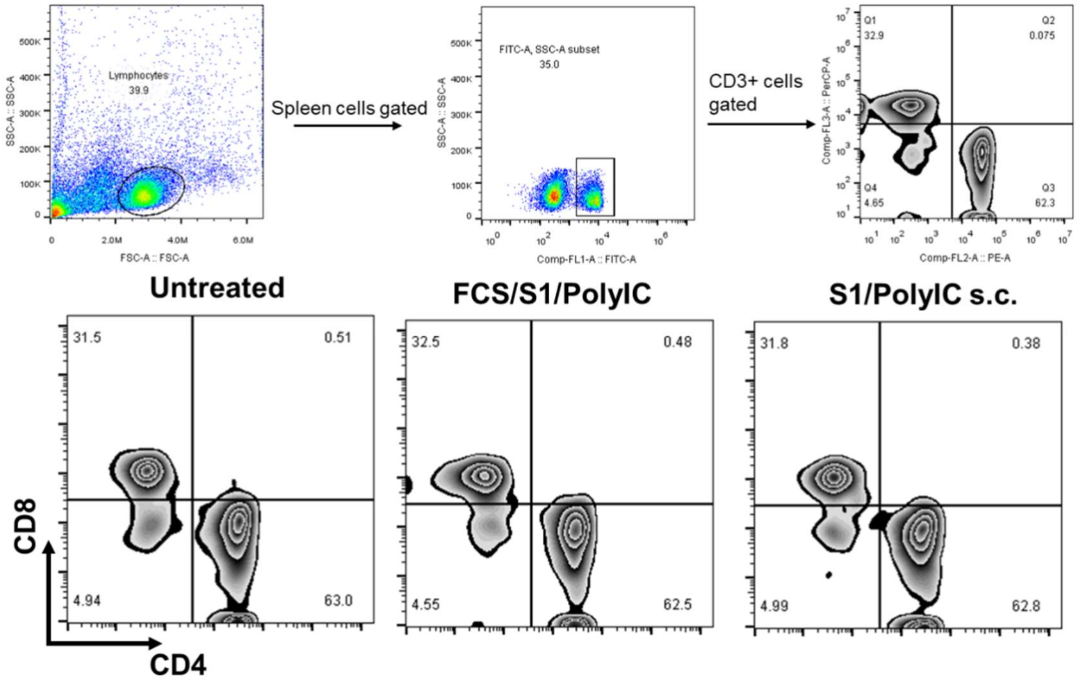

Figure S22. Representative flow cytometric plots of (Figure 5g) $CD4^+$ T cells and (Figure 5h) $CD8^+$ T cells in spleens after different administrations on day 28.

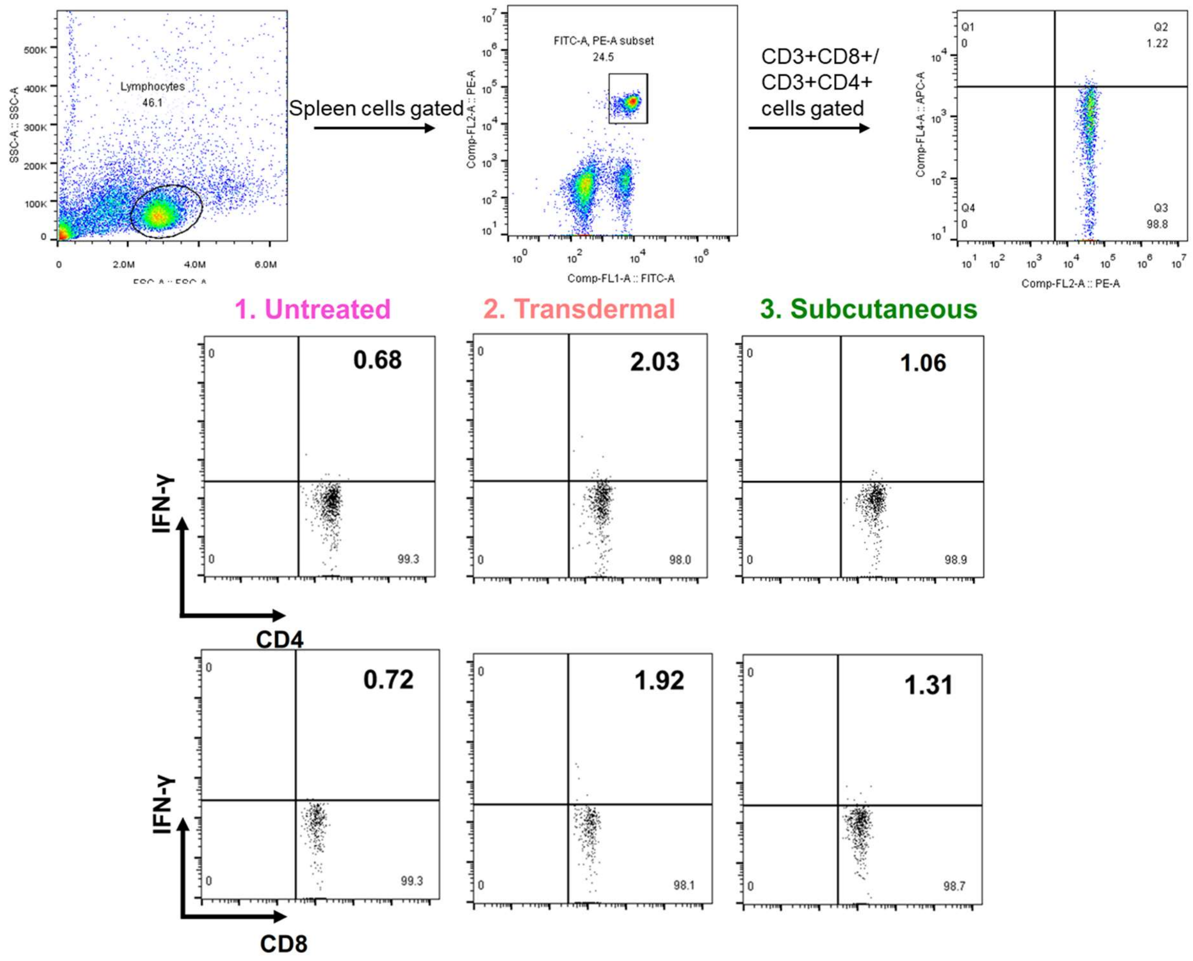

Figure S23. Representative flow cytometric plots of (Figure 5i) IFN-γ^+ $CD4^+$ T cells and (Figure 5j) IFN-$\gamma^+$$CD8^+$ T cells in spleens after different administrations on day 28.

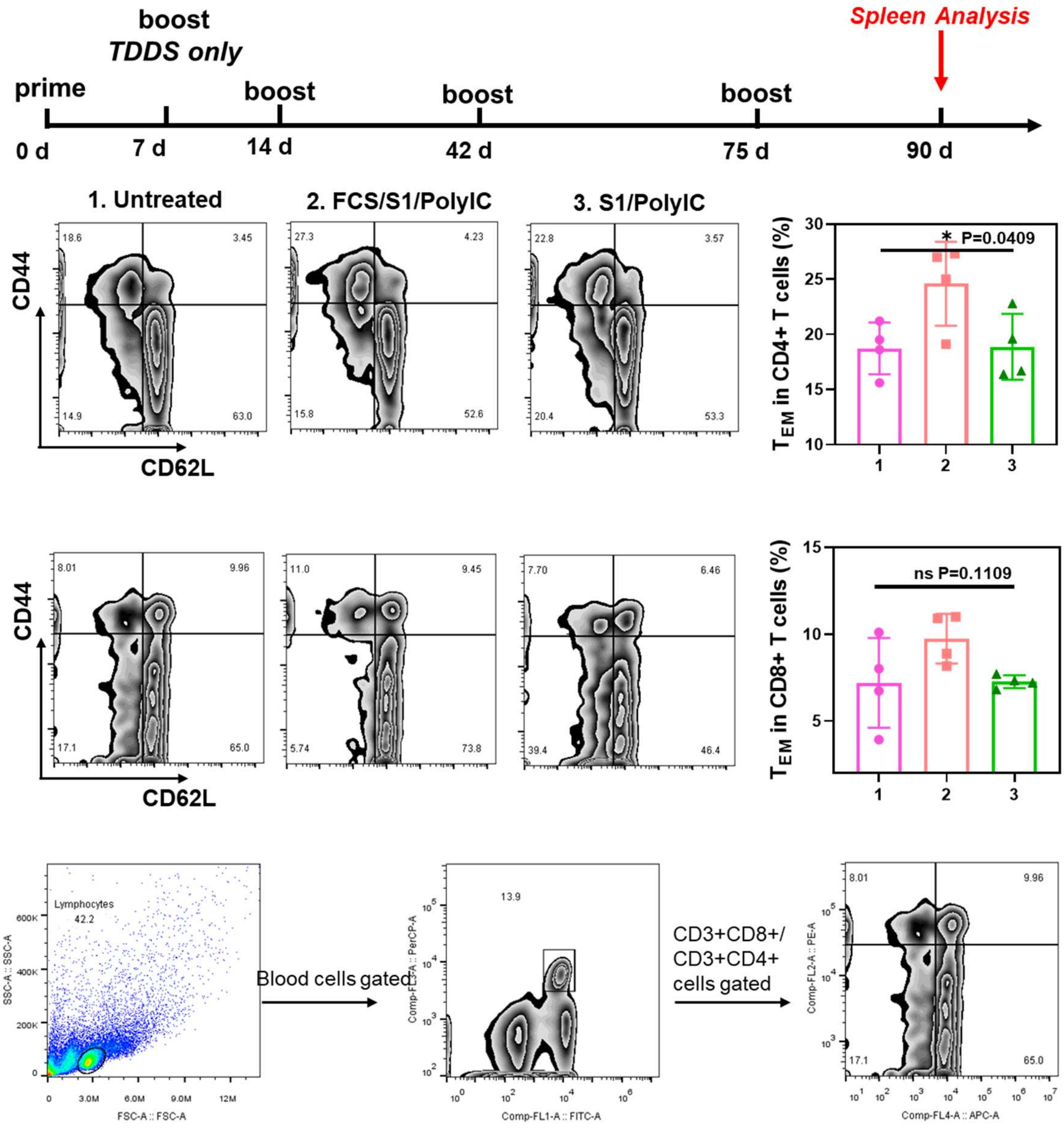

Figure S24. The long-term adaptive immune effect of mice after different vaccination administrations. Splenic lymphocytes were collected 90 days post administrations and stained with FITC-CD3, PE-CD44, APC-CD62L and PERCP-CD4 or PERCP-CD8. The CD44+CD62L- cells in both CD4+ and CD8+ T cells are effector memory CD4+ and CD8 T+ cells, respectively. Data are presented as mean ± standard deviation (n=4). Statistical significance was calculated via one-way ANOVA with a Tukey post-hoc test. *P < 0.05; **P < 0.01; ***P < 0.001.

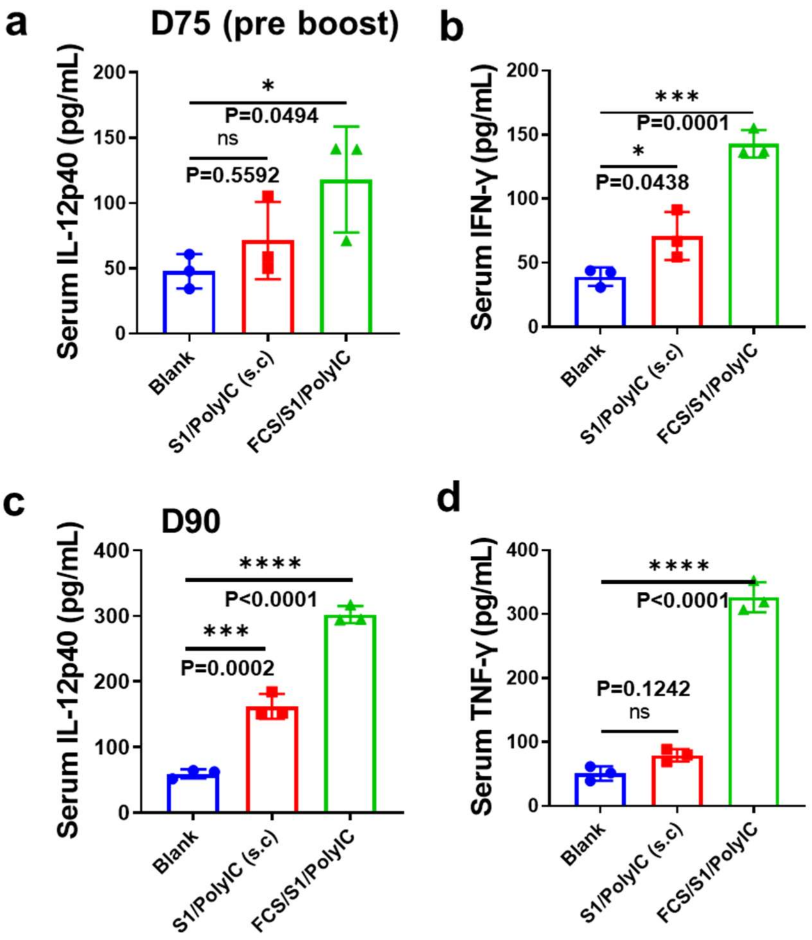

Figure S25. The sera cytokine level of IL-p40 and IFN- γ on (a&b) day 75 pre boost and (c&d) day 90. Data are presented as mean ± standard deviation (n=3 or 4). Statistical significance was calculated via one-way ANOVA with a Tukey post-hoc test. *P < 0.05; **P < 0.01; ***P < 0.001; ****P < 0.0001.

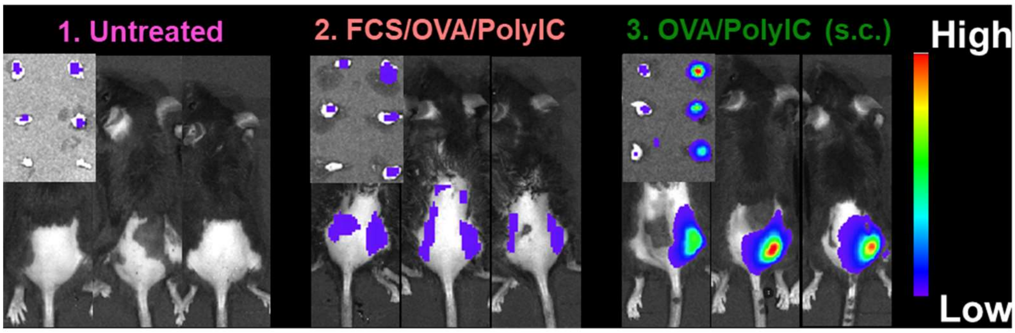

Figure S26. Representative in vivo and ex vivo fluorescent images of OVA-Cy5.5 in lymph nodes with different administration routes. It was illustrated that the FCS-based subcutaneous group showed relatively high lymph node accumulation at 24 hours post administration (n=3).

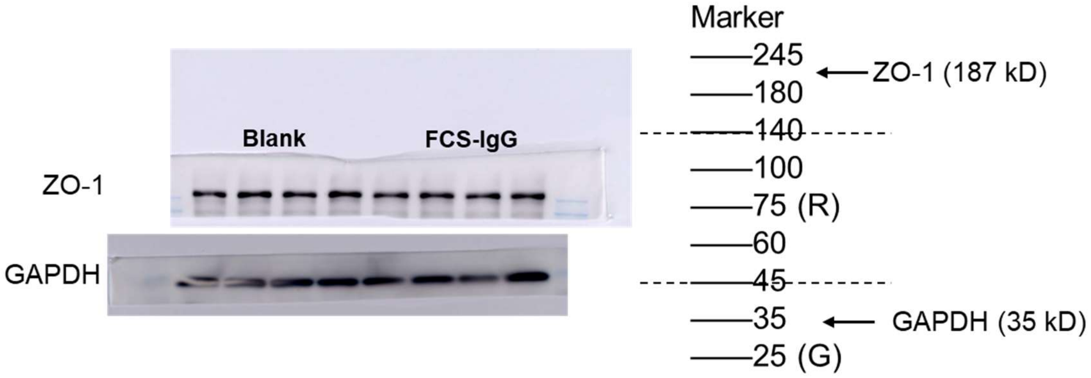

Figure S27. Raw Western Blotting figures of ZO-1 and GAPDH expression in HACAT cells with or without FCS/IgG treated (n=4).

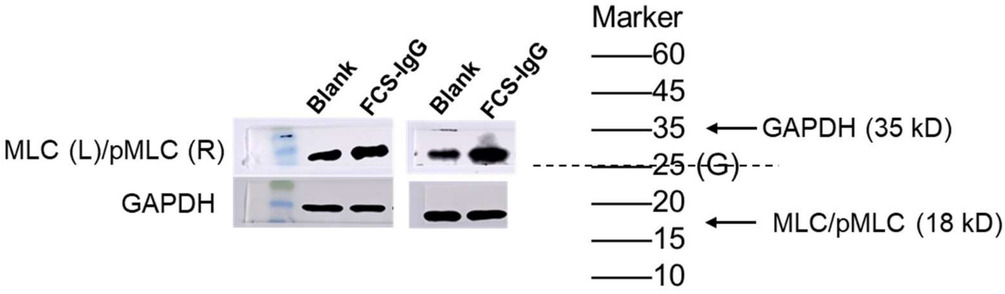

Figure S28. Raw Western Blotting figures of MLC, pMLC and GAPDH expression in HACAT cells with or without FCS/IgG treated (n=1).

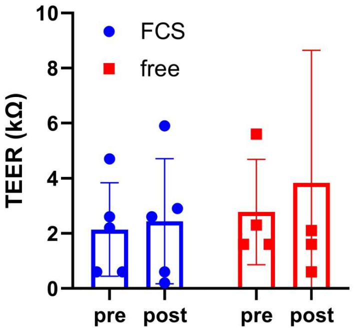

Figure S29. The skin resistances of rabbit skins pre and post transdermal delivery of FCS/IgG (n=4).

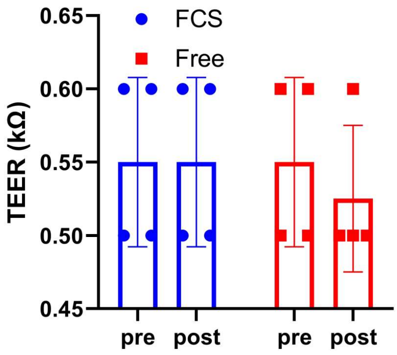

Figure S30. The skin resistances of porcine skins pre and post transdermal delivery of FCS/IgG. Data are presented as mean ± standard deviation (n=4). Statistical significance was calculated via one-way ANOVA with a Tukey post-hoc test. *P < 0.05; **P < 0.01; ***P < 0.001.
